# Supplementary material for: Patterns of Intron Gain and Loss in Fungi
Source: PLoS Biol. 2004 Nov 30;2(12):e422. doi: 10.1371/journal.pbio.0020422 (PMC532390; doi:10.1371/journal.pbio.0020422)
Supplement: Table S1 — Also available at http://genes.mit.edu/NielsenEtAl/. (4.3 MB ZIP). [file pbio.0020422.st001.zip › NielsenEtAl/html/637.html]

AN5672.1.NCU09034.1.MG08317.1.FG09883.1


```
 CLUSTAL W (1.82) Multiple Sequence Alignments - Introns Inserted


Sequence 1: NCU09034.1	410 aa
Sequence 2: MG08317.1	390 aa
Sequence 3: FG09883.1	447 aa
Sequence 4: AN5672.1	439 aa
Alignment Length: 463 aa
Number Identitical Residues: 250 aa
Alignment Score (without introns) 12004


MG08317.1 	MTSVKEFPTIKAVRSFVIGGVGSG~GDYHN~VEGGHW2H---------------------
NCU09034.1	MATARQFPTIKSIRSFVIGGVGSG~GDYHN~VKGGHW~Y---------------------
FG09883.1 	MSSVKDFPKIKAIRSFIIGGVGSG1GDYHN~VKGGHW2LIDSDISTPASKWEQYKKSRTS
AN5672.1  	MATAT-FPRIKTIRTFVIDGVGSG~GDYHN0VKGGHW~LIDSDISTPMTRWAQYRGSRTS
          	*::.  ** **::*:*:*.***** ***** *:****   .:. ::. :   .   : ::

MG08317.1 	---------------------------R~P1PACWLTHQHFERFLIGAD~PRRTNHLFEQ
NCU09034.1	---------------------------G1P~PACWLVHQHFERFLIGAD~PRNTNHLFEQ
FG09883.1 	WGINVLGSFLVEIEATDGTVGFATGFGG1P~PACWLVHQHFERFLIGAD~PRNTNLLFEQ
AN5672.1  	WGINVLGSFCVEIEATDGTKGFATGFGG~P~PACWLAHQHFERFLIGAD1PRDVNELFEK
          	 . .  .:   . .::..: . ::. .  * *****.************ ** .* ***:

MG08317.1 	MYRASMFYGRKGLPIAVISVIDLALWDLLGKIRKEPVYQMIGGATKDRLNFYCTGPEPTA
NCU09034.1	MYRASMFYGRKGLPVAVISVIDLALWDLLGKIRNEPVYKMIGGATKERLNFYCTGPDPPA
FG09883.1 	MYRASMFYGRKGLPIAVISVIDLALWDLLGKVRNEPVYRLIGGATKERLDFYCTGPEPTA
AN5672.1  	MYRGSMFYGRKGLPIAVISVIDLALWDLVGKIRNEPVYKLIGGATRSRLDFYCTGPQPGS
          	***.**********:*************:**:*:****::*****:.**:******:* :

MG08317.1 	AREMGFWGAKVPLPYCPDEGHVGMKKNIEFLRKHREAVGPDFPLMVDCYMSLNVSYTIEL
NCU09034.1	AKEMGFWGAKVPLPYCPEEGHVGLQKNIEFLRKHREAVGPNFPLMVDCYMSLNVSYTIEI
FG09883.1 	AKAMGFWGGKVPLPFCPDDGHEGLRKNVEFLRKHREAVGPDFPIMVDCYMSLNVSYTIEL
AN5672.1  	AKKAGFVGAKVALPHGPDEGVEGLLKNIEYLRKQREAVGPNFPLRVDCYMSLNVPYTIEL
          	*:  ** *.**.**. *::*  *: **:*:***:******:**: *********.****:

MG08317.1 	AEKCKD--LDINWWEECLSPDDTDGFEQIKRAHPTLKFTTGEHEYSRFGFRKLVEGRNLD
NCU09034.1	VEKTKD--LDINWWEECLSPDDTDGFEQIKRAHPTVKFTTGEHEYSRYGFRKLIEGRNLD
FG09883.1 	VKACLD--LNINWWEECLSPDDTDGFALIKRAHPTVKFTTGEHEYSRYGFRKLVEGRNLD
AN5672.1  	VKRAEQEGLHIDWWEECLSPDDFDGQALLKRAHPTVKFTTGEHEYSRFGFRKLVEGRNVD
          	.:   :..*.*:********** **   :******:***********:*****:****:*

MG08317.1 	IIQPDVMWLGGLTELLRVSAMAAAYDIPVVPHASGPYSYHFVMSQPNTPFQEYLANSPDG
NCU09034.1	IIQPDVMWLGGMTELLRVAAMAAAYDIPVVPHASGPYSYHFVMSQPNTPFQEYLANSPDG
FG09883.1 	IIQPDVMWLGGLTELLKVAALAAAYDVPVVPHASGPYSYHFQISQPNTPFQEYLANSPDG
AN5672.1  	IIQPDVMWLGGLTELLKVSALAAAYDIPVVPHASGPYSYHFVVSQPNTPFQEYLANSADG
          	***********:****:*:*:*****:************** :**************.**

MG08317.1 	KSVLPVFGDLFLDEIIPSKGFLTPKDLDQPGFGLTLNPAARAKMIPGEFWVNPAPLKALT
NCU09034.1	KSVLPVFGDLFLDEPIPTKGFLTTADLNKPGFGLTLNPVARAKLIPSAYLLAPPPMAPLT
FG09883.1 	KSVLPVFGDLFIDEPIPTKGYLTTADLDKPGFGLTINPAARAKLIPSDYLFKVPEIPQNL
AN5672.1  	HTVEPVFGNLFTNEPIPTKGYLDVSILDKPGFGLELNPSA--PLIPAASFLNPAPARSLP
          	::* ****:** :* **:**:*    *::***** :** *   :**.   .  .      

MG08317.1 	-----SGAP-AEEKK--------------EG~AVEAVVNKVQELTTS---
NCU09034.1	PVDASSGVTGAEETKPAPAPGVTEHKEESDG~VLDGFVARIQELTSL---
FG09883.1 	S----TGKEIKSNEQP----------DKPNG~TLDSLAAKVESLTTSTSS
AN5672.1  	-------APATEEKT------------ETNA1AFLDTCCEGQRLSESV--
          	           .:              ..:. ..     . : *:
```
